# Lateral Transpsoas Fusion: Indications and Outcomes

**DOI:** 10.1100/2012/893608

**Published:** 2012-11-06

**Authors:** Vishal C. Patel, Daniel K. Park, Harry N. Herkowitz

**Affiliations:** ^1^Orthopaedic Spine Fellow, Department of Orthopaedic Surgery, William Beaumont Hospital, 3601 West 13 Mile Road Royal Oak, MI 48073, USA; ^2^Orthopaedic Spine Attending, Department of Orthopaedic Surgery, William Beaumont Hospital, 3601 West 13 Mile Road Royal Oak, MI 48073, USA; ^3^Orthopaedic Spine Attending, Chairman of the Department of Orthopaedic Surgery, William Beaumont Hospital, 3601 West 13 Mile Road Royal Oak, MI 48073, USA

## Abstract

Spinal fusion historically has been used extensively, and, recently, the lateral transpsoas approach to the thoracic and lumbar spine has become an increasingly common method to achieve fusion. Recent literature on this approach has elucidated its advantage over more traditional anterior and posterior approaches, which include a smaller tissue dissection, potentially lower blood loss, no need for an access surgeon, and a shorter hospital stay. Indications for the procedure have now expanded to include degenerative disc disease, spinal stenosis, degenerative scoliosis, nonunion, trauma, infection, and low-grade spondylolisthesis. Lateral interbody fusion has a similar if not lower rate of complications compared to traditional anterior and posterior approaches to interbody fusion. However, lateral interbody fusion has unique complications that include transient neurologic symptoms, motor deficits, and neural injuries that range from 1 to 60% in the literature. Additional studies are required to further evaluate and monitor the short- and long-term safety, efficacy, outcomes, and complications of lateral transpsoas procedures.

## 1. Background

Spinal fusion has been used extensively in the thoracolumbar spine for tumors, spinal instability, deformity, and stenosis. Recent developments and advancements in minimally invasive spine surgery have created new technologies that can help avoid the morbidity of traditional open anterior or posterior surgery. Anterior surgery has been associated with complications with its approach, which include vascular complications, retrograde ejaculation, postoperative colonic obstruction, lymphocele, or injury to the sympathetic chain [1–3]. Posterior surgery, for example, posterolateral fusions, posterior lumbar interbody fusions, and transforaminal lumbar interbody fusions, have been associated with paraspinal muscle denervation, dural tears, and neural complications, such as radiculitis from malpositioned screws or retraction during the surgery to allow placement of the intervertebral cage [4–7]. 

As an alternative to anterior and posterior surgery, lateral interbody fusion was described by Pimenta in 2001 as a minimally invasive procedure for the management of lumbar spine disease [8]. Lateral interbody fusion (XLIF: NuVasive, Inc., San Diego, CA ARIA: Stryker, Inc., Kalamazoo, MI, COUGAR: Depuy Spine, Inc., Rynham, MA Ravine: K2 M, Inc., Leesburg, VA DLIF: Medtronic, Inc., Minneapolis, MN Transcontinental: Globus Medical Inc., Audubon, PA) is performed through a lateral, retroperitoneal, transpsoas approach to the disc space. Key to this approach is real-time neuromonitoring to ensure safe passage through the psoas muscle, avoiding the nerves of the lumbar plexus [9–13]. Potential benefits of the lateral approach compared with anterior and posterior approaches include the avoidance of vascular, visceral, and sexual dysfunction complications sometimes experienced in open anterior procedures, and paraspinal denervation, dural tear, and neural injuries in posterior approaches. As with anterior lumbar spine approach, the lateral approach capitalizes on the larger surface area available for fusion compared to a posterolateral fusion. In contrast, however, the anterior and posterior longitudinal ligaments remain intact, providing inherent stability during the formation of bone in fusion. 

## 2. Indications

The original indication for lateral interbody fusion delineated by Ozgur et al. was for patients with low back pain associated with degenerative disc disease but without severe central canal stenosis. In the original description of the procedure, Ozgur et al. described the contraindications to the procedure as being patients with significant central canal stenosis, significant rotatory scoliosis, and moderate to severe spondylolisthesis. However, recent reports have utilized lateral interbody fusion in conjunction with posterior instrumentation for those previous contraindications [14–16].

**Figure 1:**
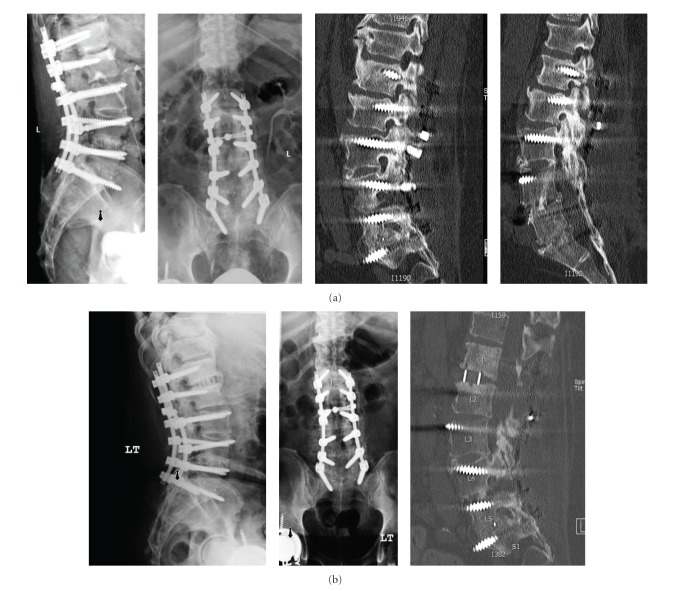
(a) Preoperative X-rays demonstrating nonunion at L1-2 after posterior instrumented fusion and decompression from L1-S1. (b) Postoperative X-rays demonstrating XLIF at L1-2.

Current indications include degenerative disc disease, spinal stenosis, degenerative scoliosis, nonunion, trauma, infection, and spondylolisthesis (grade I or II) [9, 14, 16–20]. Some of these indications also require posterior fixation.  Figure 1 is an example of an XLIF performed at L1-2 for a nonunion at the proximal aspect of a long adult deformity.

Contraindications to this technique for standalone applications include severe spinal stenosis, vascular abnormalities, and significant spondylolithesis. Relative contraindications include previous retroperitoneal surgery and severely collapsed disc spaces [21, 22].

## 3. Technique

**Figure 2:**
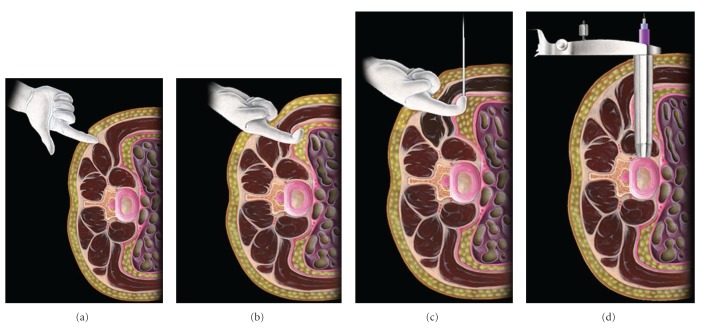
Two incision technique for a lateral transpsoas approach. (a) Surgeon's Finger traversing paraspinal muscle incision site, (b) finger identifying the retroperitoneal space, (c) surgeon's finger guiding the first dilator onto the psoas major, and (d) dilator in place traversing the psoas major directly over the intended intervertebral disc space (image reprinted with permission from Nuvasive Inc., San Diego, CA).

The lateral procedure, as originally described by Ozgur et al. and demonstrated in Figure 2, is performed under general anesthesia with the patient in the lateral decubitus position on a radiolucent table. Preoperative evaluation of the spine and vascular anatomy on imaging dictate a right or left lateral decubitus approach. Neuromonitoring is essential for this approach due to the lumbar plexus anatomy in the psoas. Because monitoring is needed, paralytic anesthetics must be avoided during the approach. Once the patient is positioned, intraoperative fluoroscopy is used to obtain a perfect lateral and AP radiograph. Next using blunt dissection, the retroperitoneal space is entered using one or two incisions. Using a series of sequential dilators, the psoas is entered down to the center of the disc space. During this exposure, neuromonitoring is used to ensure the safety of the working channel. Discectomy and disc space preparation are then performed using standard techniques with a combination of pituitary rongeurs and ringed curettes under direct visualization. After complete preparation of the disc space, an intervertebral cage that spans the space with a wide aperture that is prefilled with bone graft is inserted into the disc space between the two end plates. The external oblique fascia, subcutaneous layer, and skin are then closed.

## 4. Results and Complications

One of the earliest series of patients that underwent a lateral approach was reported by Rodgers et al. in 2007 [17]. Indications for surgery were for various degenerative conditions. They reported the procedure was safe and reproducible with a low complication rate of 2% overall, with no major complications. Rodgers et al. noted a decrease in the VAS pain scores of 68%. In another series, Knight et al. in 2009 reported on 58 patients who underwent a lateral interbody arthrodesis for degenerative disc disease [23]. Compared to Rodgers et al, they reported longer operative times, mean of 161 minutes, and a higher complication rate, 22.4% overall. Of the 13 patients who experienced complications, 9 of them were approach related with ipsilateral L4 nerve root injury in two cases, irritation of the lateral femoral cutaneous nerve in 6 patients and significant psoas muscle spasm that required extended hospitalization in two patient. Of the four other complications, three were medical and one was an acute subsidence of the implant. Rodgers et al. published another series on 100 patients who underwent XLIF for adjacent level degeneration adjacent to a prior spinal fusion surgery with similar improvement in VAS as their previous report. They reported nine complications for a total complication rate of 9%, with two patients each having postoperative urinary retention, cardiac complications, and ileus, one patient having transient tibialis anterior weakness that resolved in two weeks, one nonunion, and one vertebral body fracture. Of note, one patient had transient thigh symptoms postoperatively, which they did not count as complications [18]. Berjano et al. recently published their results of 97 patients who underwent lateral interbody fusion for a variety of indications, most commonly degenerative disc disease in 78 patients [24]. They reported no permanent neurological, vascular, or visceral injuries. Transient neurological symptoms were present in 7% of cases, though they all resolved within 1 month from surgery. Transient thigh discomfort was observed in 9%, and the overall complication rate was 12%. Clinical success was reported in 90% of the patients at six months postoperatively. Ozgur et al. published one of the first studies with a two-year followup for lateral interbody fusion in 62 patients. They reported a 19% minor complication rate, though a significant number of patients had transient hip flexion weakness and upper thigh numbness that resolved in most by six weeks. Functional improvements were maintained out to two years of followup [25]. 

The previous reports included patients with various degenerative conditions. As the lateral approach has gained more acceptance into the spine community, newer and more specific indications have been found. One of these indications is adult degenerative scoliosis. Historically, the surgical treatment for adult degenerative scoliosis has been associated with significant complications, including neurologic deficits, pulmonary embolism, infection, and death. Complication rates have reached as high as 30% in older patients. Utilizing an anterior approach for adult degenerative scoliosis, Daubs et al. reported a 10.9% incidence of vascular injury [20]. Isaacs et al. presented the first large multicenter series using a minimally invasive approach in the treatment of adult scoliotic deformity with 107 patients with 75.7% percent of the cases including posterior supplemental instrumentation with 64.2% of those cases placed using minimal access posterior surgical techniques; 35.8% using standard open techniques, and the rest without posterior surgery. They noted a major complication rate of 12.1% overall, with no vascular complications observed [26]. Of the major complications, two were medical with one case of an MI and the other sepsis. The other twelve major complications were surgical, with one case of a kidney laceration, 3 wound infections, 1 DVT, and 7 motor deficits. Furthermore, 36 patients (33.6%) reported weakness after surgery; and of those, 29 reported hip weakness that the authors attributed to the transpsoas approach. Fortunately, 86.2% of the patients with weakness had resolution by six months of followup. Predictor of any major complication was strongly correlated to the number of levels of surgery. Interestingly, patients that underwent an isolated minimally invasive lateral interbody fusion had fewer major complications in the perioperative period than those undergoing supplemental open posterior fusion.

In addition, Anand et al. reported on 12 patients who underwent combined posterior instrumentation and minimally invasive procedures for the treatment of adult degenerative scoliosis that included lateral interbody fusion [14]. The study reported pain reduction of 32.4%, though a lower blood loss than historically reported for traditional scoliosis surgeries. Furthermore, Anand et al. reported 25% of the patients with dyesthesias over the thigh, and even one patient with quadriceps weakness, though they all resolved within six weeks. In a longer follow-up paper (22 months), Anand et al. published on 28 patients with degenerative scoliosis. They found continued significant improvement in VAS pain (57%) and ODI functional outcome (82.1%) scores. Of note, they found that the incidence of thigh discomfort and numbness in up to 74% of the patients, though overall, 100% of the patients maintained correction of their deformity with verification of a solid fusion on radiographs at last followup [15]. Lastly, Dakwar et al. reported similar results in a series of 25 adult deformity patients with mean 11-month followup [16]. Despite the fact that 24 of the patients underwent multiple level lateral interbody fusion, their reported mean operative time was short at 108 minutes, with minimal mean blood loss of 53 mL. They reported a complication rate of 24% overall, with 12% of the patients experiencing transient postoperative anterior thigh numbness ipsilateral to the side of approach in the distribution of the anterior femoral cutaneous nerve. The patients had a mean improvement of 5.7 point in the VAS and 23.7% in the ODI. Clinical outcomes reported included 70.4% and 44.2% improvement in pain (VAS) and function (ODI), respectively. Of the 25 patients, 20 had minimum 6-month followup, all of whom had evidence of spinal fusion on CT scan or flexion/extension radiographs.

As stated previously, another indication of lateral interbody fusion is for indirect decompression of the spinal canal and neuroforamen. Oliveira et al. performed a lateral interbody at 43 levels in 21 patients with the primary diagnosis of lumbar stenosis with degenerative disc disease and grade I or II spondylolisthesis with good preliminary results [27]. They found the central and foraminal decompression was statistically significant, with an average 41.9% increase in disc height, 13.5% increase in foraminal height, 24.7% increase in foraminal area, and 33.1% increase in central canal diameter. Of note, two of the 21 patients needed additional posterior decompression as their symptoms of stenosis continued postoperatively. Elowitz et al. reported similar results in their series of 25 spinal stenosis patients with instability who underwent lateral transpsoas interbody fusion without laminectomy [28]. Their radiographic evaluation found a statistically significant increase in dural sac dimension of 54% in the anterior-posterior plane and 48% in the medial-lateral plane. Unlike Oliveira et al., they also evaluated clinical parameters, and found a statistically significant decrease in the ODI. Lastly, Kepler et al. analyzed pre- and postoperative CT scans in 29 patients who underwent lateral interbody fusion through a lateral transpsoas approach [29]. They found an average increase in the foraminal area of 35%, with posterior intervertebral height increasing 70%. Significant improvement was seen in SF-12 and ODI scores, but these improvements did not correlate significantly with increases in the foraminal areas, which they concluded reflected the multifactorial nature of symptom improvement.

An additional complication recently reported in the literature is that of an incisional hernia in a 75-year-old patient who underwent a one level XLIF [30]. While incisional hernias are a common complication of anterior abdominal surgery, with incidence rates from 2 to 14% [31], it had previously never been described as a complication of XLIF. Galan et al. recommended placing the surgical incision for XLIF as far posterior as possible into the thicker transversalis fascia, and then repairing the fascia with nonabsorbable suture at the end of the case. 

Ultimately, the most common and serious complication following a lateral transpsoas approach is postoperative thigh symptoms, that range from numbness of the thigh to frank motor deficits. The rate of thigh symptoms as shown in Table 1 ranges from 1% [18] to 60% [15]. The symptoms generally resolves weeks to months after the surgery.

## 5. Conclusion

Lumbar fusion has been shown to be clinically and cost effective for the treatment of instability, lumbar spondylotic disease, disc degeneration or herniation, facet degeneration, spondylolisthesis, stenosis, or scoliosis [21, 32]. Despite its benefits, both anterior and posterior surgeries have significant complications, which include the potential for vascular and neurologic injury, deep vein thrombosis, wound complications, infection, and even death. The open posterior approach to the lumbar spine for decompression and supplemental fixation requires extensive dissection of the posterior paraspinal musculature and has been shown to lead to permanent denervation of the muscles in rats [33–35] and chronic incisional pain. There have been reports as well that have shown higher infection rates with the open posterior approach compared to minimally invasive approaches [35]. 

Due to the disadvantages of the traditional approaches, minimally invasive approaches have been developed, of which include lateral interbody fusion. Initially, lateral interbody fusion was indicated for the treatment of degenerative disc disease without significant central canal stenosis, spondylolisthesis or rotatory scoliosis. However, as surgeons have become more adept at this procedure, the indications have broadened to encompass many more pathologies. Before definitive conclusions can be made, longer term followup will be needed, but it certainly does appear promising.

## Figures and Tables

**Table 1:** Summary of published neurologic XLIF complications.

	Transient neurologic symptoms	Motor deficit
Anand et al. [14]	25%	Not recorded
Anand et al. [15]	60%	Not recorded
Rodgers et al. [17]	1%	Not recorded
Knight et al. [23]	9%	3%
Rodgers et al. [18]	1%	Not recorded
Isaacs et al. [26]	27%	33.6%
Dakwar et al. [16]	12%	Not recorded
Berjano et al. [24]	7%	Not recorded
Youssef et al. [36]	Not recorded	1%
